# Assessment of the quality and reliability of public health communication about hypertension on short video platforms in China

**DOI:** 10.3389/fpubh.2026.1820704

**Published:** 2026-08-11

**Authors:** Jin Wu He, Ke Zhang, Haiyan Yang, Tianyi Zhao, Jie Wu, Liping Deng

**Affiliations:** Department of Geriatrics, The First People's Hospital of Zigong City, Zigong, China

**Keywords:** DISCERN, global quality scale (GQS), health education, hypertension, information quality, short videos

## Abstract

**Background:**

Hypertension is one of the major modifiable cardiovascular risk factors in China. Persistent and poorly controlled hypertension contributes to a wide spectrum of adverse cardiovascular outcomes, yet public awareness and understanding of the condition remain suboptimal. With the rapid expansion of digital media, social platforms have emerged as important channels for disseminating health information. This study evaluates the quality of hypertension-related content presented in short-video formats on widely used social media platforms.

**Methods:**

Newly registered accounts were used to retrieve the top 100 videos from Douyin, Bilibili, and Kwai based on each platform’s default comprehensive ranking algorithm. The collected videos were subsequently screened following predefined criteria, which included removal of duplicates, silent clips, advertisements, irrelevant content, and non-Chinese language videos. In addition, videos produced by the same creator on the same platform were consolidated into a single entry to avoid redundancy. The reliability of the videos was assessed using the DISCERN instrument, and their overall quality was evaluated with the Global Quality Scale (GQS). A comparative analysis was further performed to examine differences in video characteristics and quality across the three short-video platforms.

**Results:**

A total of 267 videos from various creators were included in the analysis, with Douyin contributing 36.3% (140/267), Bilibili 31.1% (83/267), and Kwai 32.6% (87/267). With respect to video sources, non-specialist physicians produced the largest proportion of content (52.4%, 140/267), followed by specialist physicians (31.5%, 84/267). In the assessment of overall video quality, Bilibili demonstrated the highest completeness scores (*p* < 0.001). Douyin and Bilibili demonstrated comparable Global Quality Scores (GQS) and both performed significantly better than Kwai (*p* < 0.001). Nevertheless, all three platforms exhibited uniformly low DISCERN scores. When comparing creators with different professional backgrounds, videos produced by non-profit organizations and specialist physicians showed significantly higher overall quality than those generated by non-specialist physicians and individual users.

**Conclusion:**

Overall, the quality of the videos was suboptimal. Although hypertension-related content produced by nonprofit organizations and cardiovascular specialists achieved higher scores in overall quality, the presentation of these videos was relatively uniform. This homogeneity limited the breadth of hypertension-related knowledge that viewers could obtain from short-video platforms.

## Introduction

Hypertension is a major global health burden and a leading contributor to adverse cardiovascular events and premature mortality (1). Two principal pathogenic mechanisms are currently recognized: primary hypertension and secondary hypertension. Primary hypertension results from a complex interplay of factors, including genetic susceptibility, obesity, and excessive sodium intake (2). In contrast, secondary hypertension refers to elevated blood pressure caused by specific, identifiable conditions or external factors, such as primary aldosteronism or Cushing’s syndrome (3). The multifactorial mechanisms underlying hypertension pose substantial challenges for effective management. In many cases, monotherapy is insufficient to achieve sustained blood pressure control within safe physiological ranges. Such inadequate control increases the risk of hypertension-related complications that can severely compromise human health.

In China, substantial gaps remain in hypertension-related health education, disease management, and primary care services (4, 5). A considerable proportion of the population continues to engage in unhealthy lifestyle and dietary behaviors. Evidence indicates that adopting a low-sodium, light diet in combination with regular physical activity can significantly reduce blood pressure levels (6). However, many individuals with hypertension continue to consume diets high in sodium, fat, and sugar (7). Moreover, when hypertension has not yet caused substantial damage to vital organs such as the cardiovascular, cerebrovascular, hepatic, or renal systems, its clinical manifestations often remain subtle and easily overlooked (8). Limited awareness of hypertension—arising from multiple contributing factors—can delay timely diagnosis and treatment. Such delays may negatively influence both quality of life and overall survival. Furthermore, the asymmetry of medical information between healthcare providers and patients intensifies communication challenges (9), which may further reduce patient adherence to clinical recommendations. These issues underscore the necessity of strengthening public awareness and improving health education and management related to hypertension.

With the widespread adoption of smartphones and the accelerating pace of modern life, short videos have become a prominent medium for delivering concise and engaging content. They are rapidly emerging as a primary source of entertainment and learning for many individuals. An increasing proportion of the population now obtains health-related information through short-video social platforms (10, 11). Since the onset of the COVID-19 pandemic, hospital visits have markedly declined, further amplifying the role of digital media in health information dissemination (12). In China, short-video platforms such as Douyin, Kwai and Bilibili have attracted approximately 888 million users, and this number continues to rise (13). These platforms enable users to search for and access health information and guidance, thereby contributing to improvements in health literacy (14–16). Additionally, users can readily access relevant video content by searching specific keywords, further accelerating this trend (17). However, the quality of such content remains highly variable. Evidence suggests that the accuracy and reliability of medical information disseminated through video-based media require substantial improvement (18), and instances of misinformation may mislead the public (19, 20). A comprehensive evaluation of the quality of short videos related to hypertension health education in China is currently lacking. This study aims to assess the overall quality of hypertension-related short videos by analyzing data collected from a survey of major short-video platforms.

## Materials and methods

### Data collection

This study analyzed the top 100 videos about hypertension from Douyin, Bilibili and Kwai. Searches were performed via new user accounts using each platform’s default sorting algorithm, with no restrictions on release time, duration or scope. We excluded repetitive, silent, promotional, off-topic and non-Chinese videos.

Data collection took place at 20:04 (UTC + 8) on 22 July 2023 on an Android device with location set to Mainland China. Cache and browsing history were cleared before each search, and accounts stayed logged in throughout the process. We used official mobile apps: Douyin (26.1.0), Bilibili (7.22.0) and Kwai (11.6.20), rather than web versions, and all keywords were in simplified Chinese.

**Figure 1:**
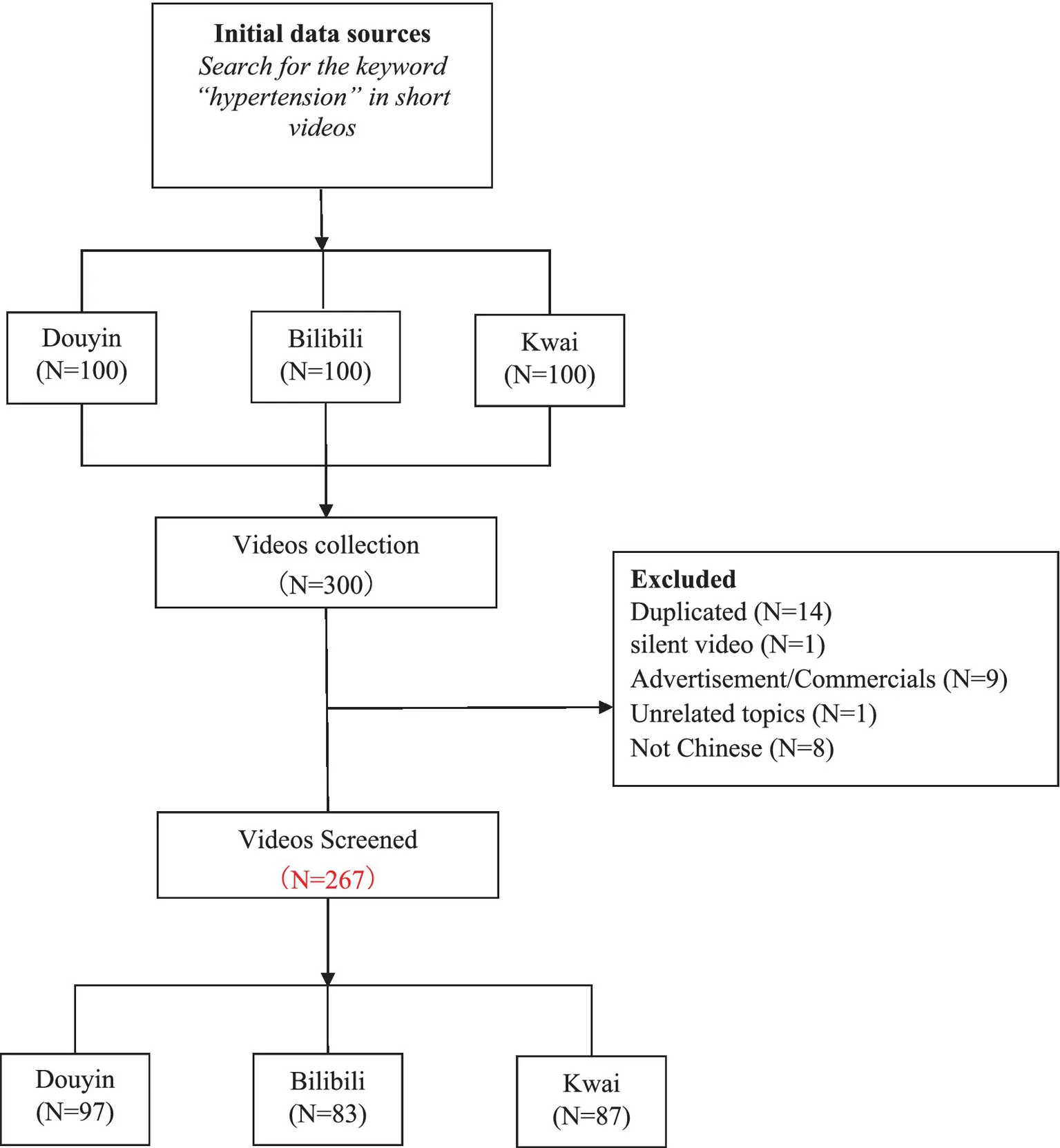
Hypertension video screening flowchart.

Three researchers completed data screening. All received standardized training with a self-developed coding manual. Two researchers collected platform data in one single session to avoid ranking fluctuations. All scoring sheets were cross-checked; disagreements were resolved via group discussion, and uncertain cases were adjudicated by a senior clinician (Figure 1). Cohen’s Kappa test showed high inter-rater reliability, with values of 0.99 for content completeness, 0.95 for GQS and 0.96 for DISCERN.

### Evaluation methods

According to the *2024 ESC Guidelines for the Management of Elevated Blood Pressure and Hypertension* (21), we divided video content into seven core knowledge domains: epidemiology, etiology, symptoms, diagnosis, treatment, prevention and complications. We adopted a structured scoring system to assess domain coverage: 0 points for no relevant content, 1 point for fragmented coverage of key points, and 2 points for systematic and comprehensive explanation of core knowledge. The sum of scores across all domains yielded an overall content integrity score, with a maximum of 14 points. The DISCERN instrument is a well-established international tool for assessing the reliability of health-related video content (22). Considering the inherent characteristics of short videos—concise information, fragmented presentation and limited duration—the original full-scale DISCERN is not fully suitable for short-video health communication. Accordingly, we simplified its items and selected five core dimensions tailored for hypertension-themed short videos: clarity, relevance, traceability, robustness and impartiality (10, 16, 23, 24). Each dimension was scored 1 point, leading to a total maximum score of 5. We further adopted the Global Quality Scale (GQS) (25) to evaluate the overall quality and patient-oriented usefulness of the videos. On this scale, a score of 1 means the content offers no practical help to patients, and a score of 2 indicates limited usefulness. A score of 5 stands for excellent quality and high value for patient education.

### Statistical analysis

Data collection and organization were completed in Microsoft Excel, while all statistical analyses were performed using IBM SPSS Statistics version 26.0 (IBM, Chicago, IL, USA). Since none of the continuous variables followed a normal distribution, they were summarized as median (interquartile range). Categorical variables were reported as counts and percentages. Between-group comparisons of continuous variables across three platforms were conducted using the Kruskal–Wallis H test, and chi-square tests were adopted to examine disparities in categorical variables. Post-hoc pairwise comparisons were subsequently implemented via the Mann–Whitney U test, with the Bonferroni correction applied to mitigate type I error arising from multiple testing. To control confounding bias, we constructed fully adjusted multivariable regression models with three primary outcome measures, namely the content integrity score, Global Quality Score (GQS), and modified DISCERN score. Ordinal logistic regression was used to analyze the GQS, as it represented an ordinal categorical variable, whereas multiple linear regression models were applied for the content integrity score and modified DISCERN score. All continuous independent variables (video duration, likes, comments, and favorites) were standardized via Z-score transformation to eliminate dimensional differences and facilitate the intuitive interpretation of regression coefficients. All statistical tests were two-tailed, and a *p*-value < 0.05 was defined as the threshold for statistical significance. All figures generated in the present study were visualized with Origin software.

## Results

### Characteristics of hypertension related-short video

We retrieved the top 100 videos from three major short-video platforms—Douyin, Bilibili, and Kwai—using each platform’s default comprehensive ranking algorithm. Following the application of the predefined exclusion criteria, a total of 267 videos from various creators were included in the final dataset. Of these, Douyin contributed 36.3% (97/267), Bilibili accounted for 31.1% (83/267), and Kwai represented 32.6% (87/267). The overall quality of the videos was low, with a median completeness score of 3 (IQR 2–4), a GQS score of 3 (IQR 2–3), and a DISCERN score of 2 (IQR 2–3). Regarding video sources, non-specialists contributed the largest proportion of content (52.4%, 140/267), followed by specialists (31.5%, 84/267). In terms of content categories, hypertension treatment was the most frequently discussed topic (65.5%, 175/267), followed by prevention (50.6%, 135/267), etiology and complications were similar, at (36.8%) and (39.0%). In contrast, symptoms (35.2%), diagnosis (32.6%), and epidemiology (11.7%) were addressed far less frequently. Overall, the videos attracted substantial user engagement, with a median of 1,525 likes (IQR 140–16,000), 570 shares (IQR 31–5,610), 618 favorites (IQR 89–3,381), and 82 comments (IQR 6–586). The median video duration was 115 s (IQR 67–237) (Table 1).

**Table 1:** Video characteristics.

Characteristic		*N* = 268
Short-video sharing platforms [*n* (%)]		
	Douyin	97 (36.3)
	Kwai	87 (32.6)
	Bilibili	83 (31.1)
Video source [*n* (%)]		
	Non-specialists	140 (52.4)
	Specialists	84 (31.5)
	Nonprofit organizations	21 (7.9)
	Individual user	22 (8.2)
Video content [*n* (%)]		
	Epidemiology	31 (11.7)
	Etiology	98 (36.8)
	Symptom	94 (35.2)
	Diagnosis	87 (32.6)
	Treatment	175 (65.5)
	Prevention	135 (50.6)
	Complication	104 (39.0)
Number of likes [median (IQR)]		1,525 (140–16,000)
Number of comments [median (IQR)]		82 (6–586)
Number of collections [median (IQR)]		618 (89–3,381)
Number of shares [median (IQR)]		570 (31–5,610)
Video duration [s, median (IQR)]		115 (67–237)
Completeness score [median (IQR)]		3 (2–4)
GQS scores [median (IQR)]		3 (2–3)
DISCERN scores [median (IQR)]		2 (2–3)

### Characteristics comparison of different sharing platforms

We analyzed hypertension-related videos from three major short-video platforms. On Douyin, videos created by cardiologists accounted for 47.4% (46 videos). The proportions dropped to 29.9% on Kwai and 14.5% on Bilibili, with statistically significant differences across platforms (*p* < 0.001). Video durations on Douyin and Kwai were markedly shorter than those on Bilibili. For interaction indicators, Douyin videos received far more likes, comments and favorites than content on the other two platforms.

Notably, likes, comments, favorites and shares are influenced by each platform’s user profile and operational rules. Thus, raw data cannot directly reflect cross-platform popularity and dissemination performance, and we interpreted all results cautiously while fully accounting for platform heterogeneity. Repost data for Kwai was incomplete and therefore excluded from statistical analyses (Table 2).

**Table 2:** Comparison of different short-video sharing platforms.

Variables	Douyin (*N* = 97)	Kwai (*N* = 87)	Bilibili (*N* = 84)	*p-*value
Video source [*n* (%)]				<0.001
Non-specialists	44 (45.4)	61 (70.1)	35 (42.2)	
Specialists	46 (47.4)	26 (29.9)	12 (14.5)	
Nonprofit organizations	6 (6.2)	0 (0)	15 (18.1)	
Individual user	1 (1.0)	0 (0)	21 (25.3)	
Number of likes [median (IQR)]	11,000 (716–43,000)	3,554 (461–15,500)	161 (39–534)	<0.001
Number of comments [median (I QR)]	353 (30–1,192)	215 (57–878)	3 (1–20)	<0.001
Number of collections [median (I QR)]	1718 (142–7,728)	1,415 (166–5,331)	158 (51–576)	<0.001
Number of shares [median(IQR)]	4,865 (153–16,000)	–	123 (26–570)	<0.001
Video duration [S, median (IQR)]	79 (55–122)	92 (72–156)	294 (135–648)	<0.001
Completeness score [median (IQ R)]	3 (2–4)	2 (2–3)	3 (2–6)	<0.001
GQS scores [median (IQR)]	3 (2–3)	2 (2–3)	3 (2–3)	<0.001
DISCERN scores [median (IQR)]	2 (2–2)	2 (1–2)	2 (2–3)	<0.001

IQR, Interquartile Range; GQS, Global Quality Scale; DISCERN, A validated instrument for assessing the quality of consumer health information; Statistical significance was set at *p* < 0.05; “–” indicates missing data.

**Figure 2:**
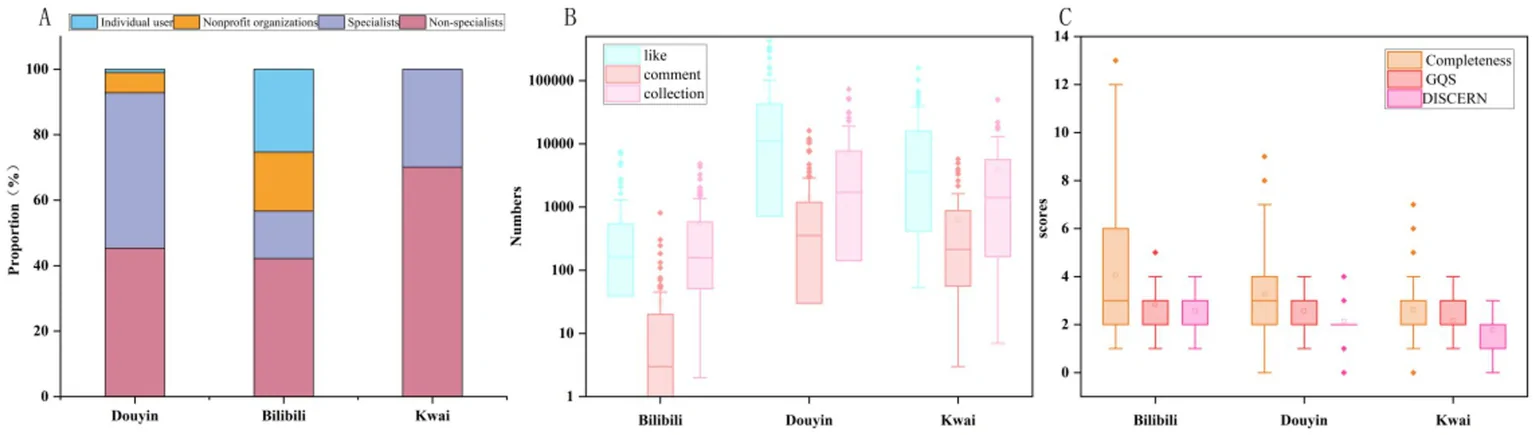
Comparison of video sources, engagement metrics, and quality scores across short-video platforms: **(A)** Stacked bar chart illustrating the distribution of video publisher identities (specialists, non-specialists, nonprofit organizations, and individual users) across Douyin, Bilibili, and Kwai. Percentages are calculated within each platform: **(B)** Box plots showing user engagement metrics, including the number of likes, comments, and favorites, for hypertension-related videos on each platform. Boxes represent the interquartile range (IQR), the central line indicates the median, and whiskers denote 1.5 × IQR: **(C)** Box plots comparing video completeness scores, Global Quality Scale (GQS) scores, and DISCERN scores across platforms. Higher scores indicate better informational completeness, overall quality, and reliability, respectively.

Regarding content performance, Bilibili videos earned the highest content integrity scores. Douyin and Bilibili had similar Global Quality Scale (GQS) scores, both significantly higher than those of Kwai. Nevertheless, DISCERN scores were generally low across all platforms, suggesting poor reliability of the hypertension-related content (Figure 2).

### Characteristics comparison of different video source

**Figure 3:**
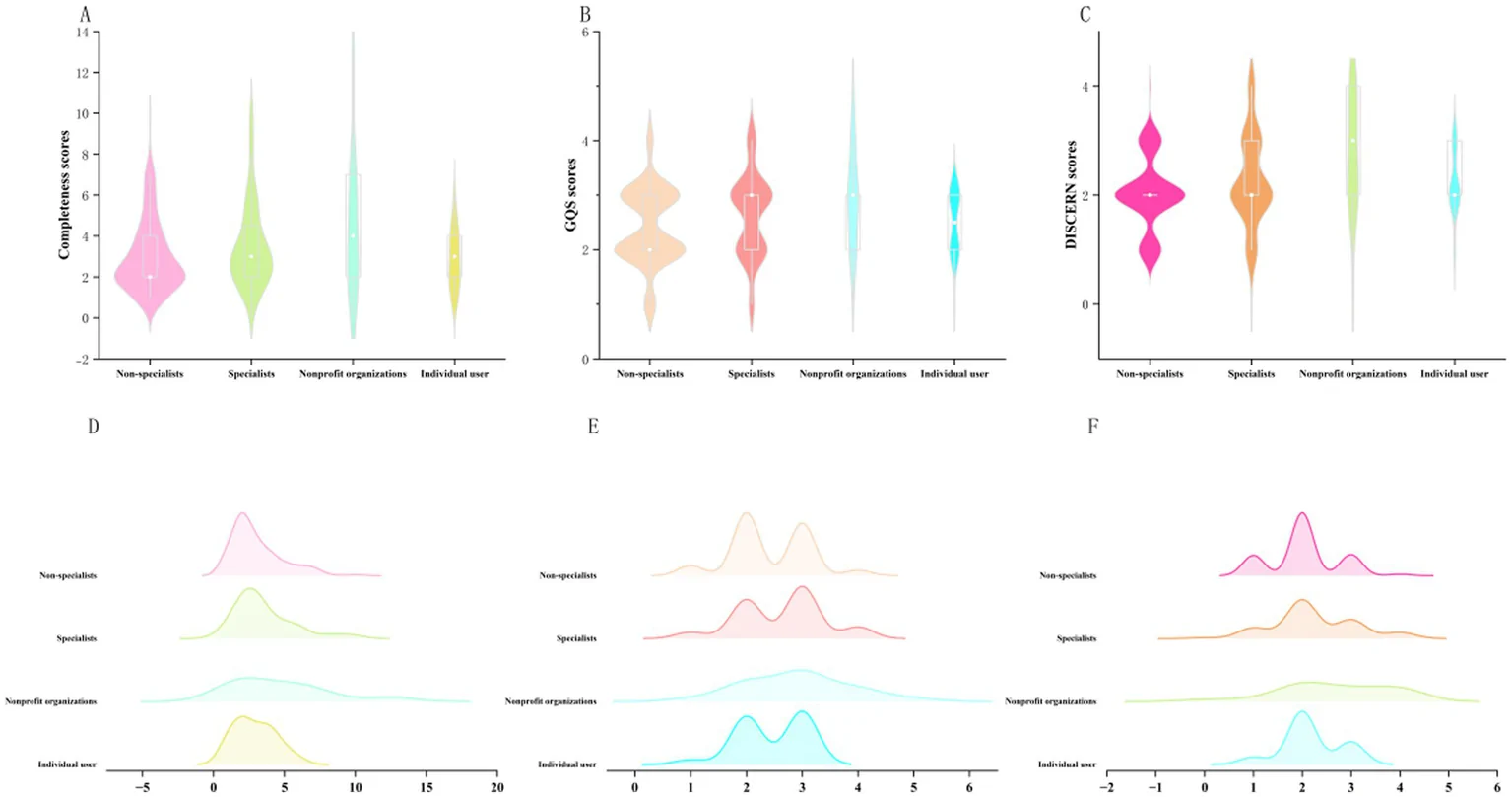
Distribution of video quality and reliability scores by publisher identity. **(A–C)** Violin plots comparing completeness scores, Global Quality Scale (GQS) scores, and DISCERN scores for hypertension-related videos published by different author types (non-specialists, specialists, nonprofit organizations, and individual users). The width of each violin represents the kernel density of the data, and the central line indicates the median score. **(D–F)** Ridge density plots illustrating the distribution patterns of completeness scores, GQS scores, and DISCERN scores across different publisher identities.

We also compared video performance across different categories of content creators. Videos posted by physicians received significantly more likes and comments than those from nonprofit organizations and individual users (*p* < 0.05). In terms of video integrity, nonprofit organizations achieved a median score of 4, outperforming all other publisher groups. For GQS ratings, nonprofit organizations again ranked highest with a median score of 3, followed closely by cardiologists; both groups scored significantly higher than non-cardiology specialists and individual users (*p* = 0.026). A similar pattern was observed for DISCERN scores (Table 3 and Figure 3).

**Table 3:** Comparison of different video source.

Variables	Non-specialists (*N* = 132)	Specialists (*N* = 71)	Nonprofit organizations (*N* = 21)	Individual user (*N* = 44)	*p-*value	
Number of likes [median (IQ R)]	2,174 (156–15,250)	3,586 (384–31,250)	49 (16–698)	496 (188–942)	<0.001	
Number of comments [median (IQR)]	122 (8–780)	178 (29–860)	1 (0–35)	7 (3–58)	<0.001	
Number of collections [median (IQR)]	951 (81–4,363)	763 (152–4,643)	100 (24–199)	486 (218–1,476)	0.013	
Number of shares [median (I QR)]	181 (25–4,858)	2,876 (254–11,750)	54 (25–413)	532 (135–1,082)	0.009	
Completeness score [median (IQR)]	2 (2–4)	3 (2–4)	4 (2–7)	3 (2–4)	0.078	
GQS scores [median (IQR)]	2 (2–3)	3 (2–3)	3 (2–3)	2 (2–3)	0.026	
DISCERN scores [median (I QR)]	2 (2–2)	2 (2–3)	3 (2–4)	2 (2–2)	<0.01	

### Influencing factors of the quality of hypertension educational short videos

Multivariable regression analysis with confounding factors adjusted revealed that short videos about hypertension popular science released on Bilibili achieved the best overall quality, which was consistent with the foregoing analytical results. Video duration was a major positive contributor to content quality. Unexpectedly, the number of likes was significantly negatively correlated with video quality, indicating that videos with high likes do not necessarily have superior science content, whereas videos triggering user interactions including comments and collections possess better quality. In terms of creator identity types, the regression results also suggested that hypertension science videos posted by individual users had significantly lower quality than those from other types of creators (Tables 4–6).

**Table 4:** Ordered logistic regression results of GQS score.

Variables	OR	SE	*Z*-value	*p*	95% CI for OR
Kwai	0.34	0.32	−3.40	<0.001	[0.19 0.63]
BiliBili	2.20	0.40	2.01	0.04	[1.02 4.87]
Specialists	1.69	0.30	1.75	0.08	[0.94 2.92]
Nonprofit organizations	0.86	0.55	−0.29	0.78	[0.29 2.50]
Individual user	0.301	0.52	−2.312	0.0208	[0.02 0.83]
Video duration	1.001	0	3.601	<0.001	[1.00 1.00]
Number of likes [median (IQR)]	1	0	−3.299	0.001	[1.00 1.00]
Number of comments	1	0	2.339	0.0194	[1.00 1.00]
Number of collections	1	0	1.422	0.155	[1.00 1.00]

**Table 5:** Multiple linear regression results of completeness score.

Variables	*β*	SE	*T*-value	*p*	95% CI for OR
Kwai	−0.36	0.28	−1.28	0.20	[−0.90 0.19]
BiliBili	0.73	0.32	2.25	0.03	[0.09 1.36]
Specialists	0.16	0.25	0.62	0.54	[−0.34 0.65]
Nonprofit organizations	0.31	0.44	0.70	0.48	[−0.56 1.17]
Individual user	−1.02	0.44	−2.33	0.021	[−1.88–0.16]
Video duration	0.00	0	7.925	<0.001	[0.001 0.001]
Number of likes	0	0	−0.96	0.34	[0.00 0.00]
Number of comments	0	0	2.01	0.046	[0.00 0.00]
Number of collections	0	0	0.194	0.85	[0.00 0.00]

**Table 6:** Multiple linear regression results of modified DISCERN score.

Variables	*β*	SE	*T*-value	*p*	95% CI for OR
Kwai	−0.29	0.11	−2.71	<0.01	[−0.51–0.08]
BiliBili	0.44	0.13	3.51	<0.01	[0.19 0.69]
Specialists	0.15	0.10	1.49	0.14	[−0.05 0.34]
Nonprofit organizations	0.10	0.17	0.56	0.57	[−0.24 0.44]
Individual user	−0.36	0.17	−2.11	0.04	[−0.70–0.02]
Video duration	0	0	4.97	<0.001	[0.00 0.00]
Number of likes	0	0	−1.04	0.30	[0.00 0.00]
Number of comments	0	0	1.31	0.20	[0.00 0.00]
Number of collections	0	0	0.50	0.62	[0.00 0.00]

For categorical covariates, Douyin was set as the reference category for platform, and Non-specialists as the reference category for creator type.

## Discussion

According to the sixth national hypertension survey conducted in China in 2018, 24.7% of the population is affected by hypertension (5). The condition is characterized by subtle or nonspecific symptoms, the need for long-term maintenance therapy, and the potential to cause multiple complications (26, 27). Moreover, some patients hold misconceptions about antihypertensive medications, believing that once treatment is initiated, the drugs must be taken indefinitely. Such misunderstandings may lead to poor adherence to prescribed therapeutic regimens (28). Such misconceptions impede effective hypertension management and may contribute to damage of target organs. In this context, strengthening public awareness through targeted educational initiatives is particularly important to promote early diagnosis and timely intervention (29). The widespread adoption of smartphones has accelerated the growth of short-video content, which has gained substantial popularity on platforms such as Douyin. This trend has created new opportunities for the dissemination of public health information, both within China and globally (30). This study analyzed data obtained from three major short-video platforms in China. A total of 267 hypertension-related videos were identified and screened to evaluate their overall quality.

Statistical analysis shows that non-specialists produce the largest proportion of videos, accounting for 52.4%, whereas specialists contribute only 31.5%. However, specialists demonstrate significantly higher reliability and quality compared with both non-specialists and individual users. Although non-specialists perform better than individual users, the difference is not statistically significant. Notably, videos produced by nonprofit organizations exhibit high overall quality, slightly exceeding that of specialist-generated content. This phenomenon may be explained by the fact that most nonprofit organizations release content through official channels and tend to produce longer-duration videos, which confers a distinct advantage in terms of quality performance (31). Individual users often lack professional medical knowledge; consequently, much of the content they produce is based on personal treatment experiences rather than evidence-based practices. As a result, the overall quality of these videos is generally low and provides limited value for individuals with hypertension. In some instances, such content may even disseminate incorrect or misleading treatment approaches.

Analysis across the seven predefined knowledge domains revealed that most videos focused heavily on lifestyle interventions for hypertension treatment and prevention, such as physical exercise and dietary adjustments. Even videos from specialists and nonprofit organizations only briefly mentioned diagnostic criteria (32) and pathogenesis (33) without in-depth elaboration. Furthermore, the indications and contraindications of the five major classes of antihypertensive drugs recommended by current clinical guidelines were rarely covered (21, 34). There were also obvious shortcomings in interpreting disease symptoms, with an inability to clearly differentiate the typical symptoms of hypertension. Overall, the included videos failed to provide comprehensive knowledge related to hypertension management. In this regard, enriching content concerning diagnostic criteria, pathophysiology, and clinical manifestations would help optimize the completeness and practical value of hypertension-related short video content.

Across the major short-video platforms, Douyin has accumulated substantially more likes, saves, and comments than Bilibili and Kwai. However, its video quality does not exceed that of Bilibili. This pattern may be explained by Douyin’s position as the most widely used short-video platform in China, which naturally drives higher user engagement. Nonetheless, the brevity of Douyin videos limits the depth of content that can be conveyed. Although Kwai holds a moderate level of popularity, the overall quality of its videos appears lower than that of both Douyin and Bilibili, a discrepancy that may be related to comparatively less stringent content management practices.

In conclusion, the overall quality of hypertension-related videos disseminated on short-video platforms is suboptimal. Only a small proportion of these videos provide comprehensive coverage of key hypertension-related knowledge. Most exhibit low DISCERN and GQS scores, highlighting substantial deficiencies in both the reliability and overall quality of hypertension health education currently available on these platforms. The low content quality is mainly attributed to three factors: most creators have no professional medical training, content production is driven by traffic gains, and content supervision remains inadequate (35–38). In contrast, videos posted by practicing physicians and non-profit organizations show distinct advantages. Physicians produce evidence-based content in strict accordance with clinical guidelines, while non-profit organizations deliver standardized health education based on public welfare principles (31, 39). Low DISCERN scores revealed problems in content accuracy and balanced interpretation of medical risks and benefits. Low GQS scores reflected weaknesses in content structure, readability and practical value.

This study is the first to conduct a comprehensive comparative analysis of hypertension-related short videos on three major Chinese platforms: Douyin, Bilibili and Kwai. We assessed content completeness based on clinical guidelines, alongside two standard tools (DISCERN and GQS) to evaluate reliability and overall quality, and systematically compared videos from different creators, including medical professionals, non-specialists, non-profit organizations and individual users. The findings offer objective evidence and references for improving health education content on short-video platforms. As an observational content analysis, this study cannot rule out confounders such as platform type, video duration and creator background. We thus used the Bonferroni correction for multiple pairwise comparisons to strengthen the validity of statistical results. When explaining cross-platform differences in video quality, we fully considered confounding factors rather than attributing score gaps solely to platform characteristics. Future research may apply stratified analysis or multivariate models to clarify the independent impact of each variable.

Our data were solely collected from Douyin, Bilibili and Kwai. We included videos from 267 distinct creators according to the default algorithmic ranking of each platform. The massive and rapidly updated content on short-video platforms means our retrieval and inclusion criteria may have introduced selection bias. Additionally, platform algorithms that prioritize certain content can skew sample composition in terms of topic distribution, content quality and audience profiles, further compromising the representativeness of our sample. Since ranking results differ by user, time and region, the generalizability of our findings is limited. Future studies may mitigate algorithm-related bias via multi-period sampling, random extraction of video IDs, or cooperation with platforms to access more complete datasets. This research focused only on Chinese short-video platforms. Divergences in media ecosystems, public health awareness and content regulations across regions mean our results may not apply to overseas platforms. We also excluded non-Chinese videos (mainly from Bilibili), which led to the loss of relevant content and may have biased the overall quality scores for Bilibili.

## Data Availability

The datasets presented in this study can be found in online repositories. The names of the repository/repositories and accession number(s) can be found in the article/supplementary material.
